# Bright molecules for sensing, computing and imaging: a tale of two once-troubled cities

**DOI:** 10.3762/bjoc.11.298

**Published:** 2015-12-29

**Authors:** A Prasanna de Silva

**Affiliations:** 1 School of Chemistry and Chemical Engineering, Queen's University, Belfast BT9 5AG, Northern Ireland

**Keywords:** blood electrolyte analyzer, luminescent PET sensing/switching, molecular logic-based computation, photoinduced electron transfer, small molecular edge detection

## Abstract

The circumstances in Colombo, Sri Lanka, and in Belfast, Northern Ireland, which led to a) the generalization of luminescent PET (photoinduced electron transfer) sensing/switching as a design tool, b) the construction of a market-leading blood electrolyte analyzer and c) the invention of molecular logic-based computation as an experimental field, are delineated. Efforts to extend the philosophy of these approaches into issues of small object identification, nanometric mapping, animal visual perception and visual art are also outlined.

## Review

### Prologue

Colombo, Sri Lanka: A civil war leads to 100,000 needless deaths. Belfast, Northern Ireland: Another civil war and another 3,500 needless deaths. Thankfully, both wars exhausted themselves after 25 years. Colombo returned to calm in 2009, while Belfast did the same in 2005. I call both these places home. I have seen good times, bad times and good times again. Through all these times, a bit of supramolecular photochemistry [1–2] kept happening and this is my story.

My paternal grandfather was the schoolteacher in our Colombo suburb and so, the value of learning was instilled into me from age zero. A sharpening of the interest opened up when my mother bought me a flood-damaged encyclopedia of science and technology. An influential high-school teacher, Errol Fernando, focussed me further towards chemistry. Due to a shortage of chemistry lecturers in the University of Colombo, the British Council sent us a Glaxo alumnus, Vincent Arkley. He not only enthralled us with personal stories of vitamin B_12_ synthesis, but was also instrumental in opening a channel to Ph.D. study at the Department of Chemistry at Queen’s University Belfast. His former protégé at Glaxo, Ron Grigg, had risen to be the Chair of Organic Chemistry at Belfast, but recruiting decent Ph.D. students to Belfast during the troubles had been hard. Ron Grigg was persuaded by Vincent Arkley to take on several Sri Lankans. I was fortunate to work with James Grimshaw who was hugely knowledgeable in electro- and photochemistry, while Ron Grigg also kept an eye out for me. My paternal grandmother’s failing health persuaded me to return to Sri Lanka in 1980, following a very happy Ph.D./postdoctoral time in Belfast. She had been one of my carers throughout my childhood and this was my chance to return the favour. Also, I had been greatly influenced by my mother’s ability to help and care for others, even to the point of sacrifice and even against opposition. Chemistry took a back seat during the next six years as I took on a carer role and as Sri Lanka descended into a very dark place. The Department of Chemistry at the University of Colombo gave me a job, which I will always be grateful for. These eventful years proved to be the crucible in which my current research directions were forged. Two of our papers from the University of Colombo were accepted by *J. Chem. Soc., Chem. Commun*. [3–4]. When my grandmother passed on, I had an international phone call. It was Ron Grigg. He offered condolences and offered me a chance to return to Queen’s. The continuing troubles had made the recruiting of decent lecturers to Belfast difficult as well. Following family conferences, I was able to return to old friendships in Belfast. There I have stayed.

### Research beginnings

At the time that I was studying for a Ph.D. in organic photochemistry [5], the field was in transition. The study of single functional groups was nearing completion and attention was shifting to the meeting of two functionalities in a photon field. One of the most influential concepts that emerged from this ferment was photoinduced electron transfer (PET) [6–7], especially following the previous realization of its central role in green plant photosynthesis. It appealed to the physicochemical side of me that PET allows one to think primarily in terms of redox potentials, while atomic details remained secondary. Also, when a lumophore was involved, the emission could be easily observed and measured. Especially in its intramolecular manifestation, a 'lumophore–spacer–receptor’ system would behave in a modular fashion [8–10] – an accurate case of molecular engineering with attendant advantages of predictive behaviour which is rare in chemical contexts. Since an electron trying to leave a receptor would be electrostatically held back by a cation held there, it was clear that PET processes could be switched ‘off’ by an externally impressed chemical command. Since PET and luminescence compete for the deactivation of the same excited state, it was equally clear that a luminescence signal could be switched ‘on’ by chemical command. Therefore, we were fortunate to be able to introduce a general design tool of luminescent PET sensing/switching [11–15], which even handled anions and neutral species besides cations [12,16].

When the chemical command comes from what is present in the molecular neighbourhood, the three-module supramolecular system takes on the role of a sensor. Since the sensor is of nanometric dimensions, the general problem of detection and measurement of the occupants of very small spaces opens up to solutions. Scientists skilled in cell biology were to take this solution to exquisite levels in the coming years [17–18]. When the chemical command is manipulated by the scientist, the three-module system and its higher versions take on the role of a miniature information processor, e.g., a logic gate [16,19–21]. Indeed, molecular sensors and logic gates are related in several ways [16]. Both of them are rooted in two (or higher) -state equilibria between free and bound forms of a molecule, so that sensors become the simplest logic gates. However, the sensor’s ability to smoothly measure small variations in a chemical concentration [22–23], which is an analogue (rather than digital) function, arises from mass action of a large population of molecules.

**Figure 1:**
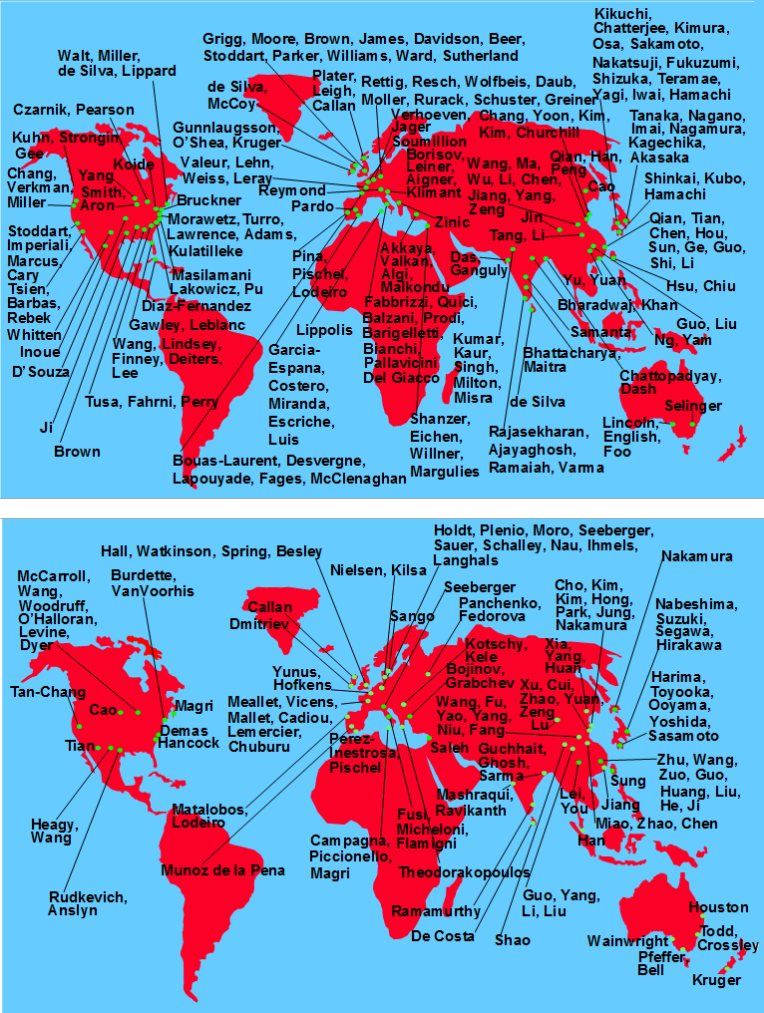
Approximate world maps of sources of fluorescent PET sensors/switches. Only the names of corresponding authors from the literature are given. Adapted from [15], Copyright (2015) The Royal Society of Chemistry.

We were able to demonstrate that the chemically switchable 'lumophore–spacer–receptor’ system naturally harnesses the diversity available in each of the three modules. For instance, the lumophore could be a fluorescent dye [3,24], a room temperature phosphor [25–26], or a lanthanide-based emitter [27–28]. Colleagues showed that even a quantum dot [29] would fit the bill. The receptor could be an amine [3,24,30], an amino acid [18,31], a crown ether [32] or a cryptand [33] and the spacer could be an oligomethylene chain [34] or nothing at all [35–36]. Since such diverse systems allow the addressing of various problems and since the design is usually straightforward, the PET sensor/switch design tool has been taken up by about 330 laboratories (Figure 1, PET maps) so far.

### A bit of medical diagnostics

**Figure 2:**
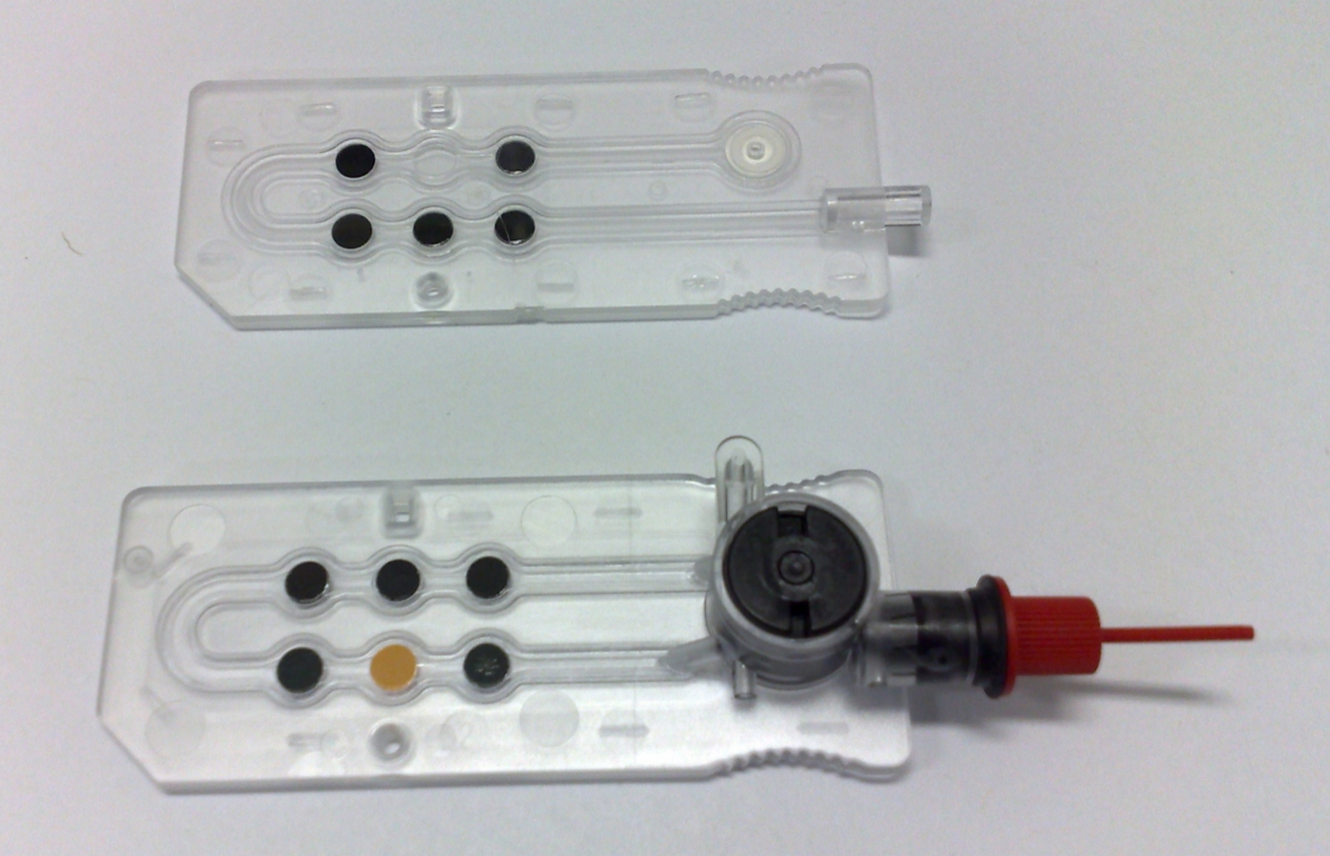
OPTI^TM^ cassettes sold by Optimedical Inc. (http://www.optimedical.com). Photograph is reprinted from [10], Copyright (2008) The Royal Society of Chemistry.

**Figure 3:**
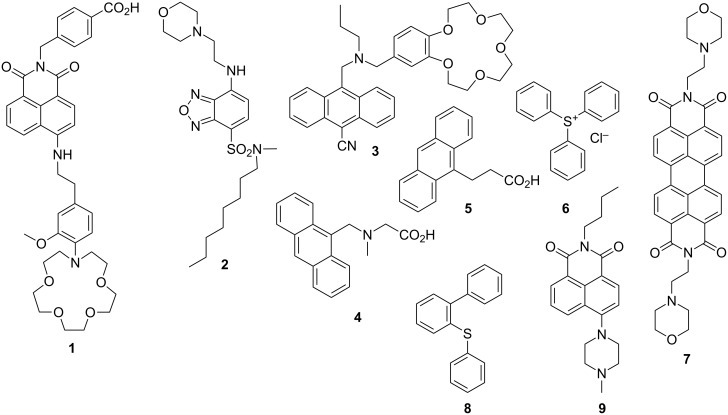
Structural formulae of the molecules discussed in this paper.

While it was clear from the beginning that fluorescent PET sensors would be useful, we saw no practical path to such development. Serendipity had to smile in the form of the interest and the commercial will of Roche Diagnostics before such a path would open. The molecular engineering capabilities of the fluorescent PET sensor design were initially put to the test to quantitatively plan an ‘off-on’ sensor for sodium in whole untreated blood. Since the normal Na^+^ level is 0.1 M, our receptor needed to have a binding strength (β_Na_) of 10 M^−1^ in neutral water. An *N*-(2-methoxy)phenylaza-15-crown-5 ether [37] fitted the bill, besides having good selectivity characteristics. PET thermodynamics were matched by the use of a 4-aminonaphthalimide fluorophore [38–40], which, in the presence of blood, also could be conveniently excited by a blue light-emitting diode once the red cells were filtered out. **1** [41] and relatives for K^+^, Ca^2+^, H^+^ and (indirectly) CO_2_ are immobilized within a millimetric channel in the chemistry module of the OPTI point-of-care analyzer (Figure 2) now sold by Optimedical Inc. [42], with sales of over $130 million thus far. An appropriately adjusted version for veterinary use is sold by IDEXX Laboratories [43] with sales of around $400 million. The structural formulae of **1** and other molecules are given in Figure 3.

A particularly touching aspect of this story occurred when Roche executives informed me that OPTI analyzers had been sold to the Sri Lanka ambulance services during the civil war there. Imagine the scene, if you will. A victim of a suicide bombing is lying in the road, fighting for life in the midst of others who have lost that fight. A paramedic identifies the sinking victim and stabilizes him/her. A sample of venous blood is drawn and analyzed in an OPTI cassette within 30 seconds. The gas and electrolyte levels in the victim’s blood are telephoned to the hospital so that a blood bag can be prepared to match the salt levels of the victim before the ambulance fights through the Colombo traffic to the Accident and Emergency ward. This precaution prevents salt shock occurring during transfusion. Before the OPTI was available, many victims died on the operating table due to salt shock even though the surgeons and nurses had followed their procedures assiduously. In other words, some of these Colombo residents (like I was) are now alive thanks to a fluorescent PET sensor.

In addition to this ex vivo application, which had an easier accreditation process through the medical watchdog institutions as compared to an in vivo version, there are emerging instances where fluorescent PET sensors are immobilized on the tips of optic fibres placed in a vein, to allow continuous monitoring of blood. Glucose monitoring [44–45] is one such success [46]. Problems of white cell attack and subsequent covering up of the fibre tip, which frustrated previous commercialization efforts along this path [47], appear to have been beaten by the use of new biocompatible hydrogel coatings. The small size of molecules allows their successful use in these millimetric spaces.

### A bit of map-making

Map-making is not the exclusive domain of cartographers and surveyors. There are sub-nanometric environments such as those bounding membranes where concentrations of species like H^+^ can be radically different to what is found in the bulk water [48–49]. Since membrane-bounded H^+^ forms the heart of the field of bioenergetics [50], these concentrations need to be located as a function of position with respect to the membrane. Such maps can be constructed by using fluorescent PET sensors equipped with extra modules for fine positioning and for reading the position, e.g., **2** [51]. The former task is achieved by using groups of various hydrophobicity to allow the sensor to reach an equilibrium position in a membrane–water interfacial region [52]. The second challenge is addressed with fluorophores which achieve charge-separated excited states [12,53] so that their interaction with the local dipoles of the neighbourhood causes shifts in the emission spectra. Simultaneous monitoring of wavelength and intensity data to obtain position and H^+^ concentration information respectively, is another example of two-dimensional fluorescence sensing [54]. Maps of local H^+^ concentration versus position show the rapid fall-off of proton levels as non-polar membrane surfaces are approached [51]. So we see that even sub-nanometric spaces can be accessed by fluorescent PET-based molecules in order to shed light on these tiny worlds.

### Emulating a bit of digital electronics

I was introduced to digital electronics in undergraduate physics classes but priceless ‘hands-on’ encounters were arranged by Satish Namasivayam. The devices came off the page of the textbook at that time and the logic gate hardware of computers lost a bit of their mystique. George Boole’s disciples had shown how the Master’s ideas concerning binary digits in communication [55] could be developed into information processors [19–20]. The modern manifestations of these processors are the logic gates cut from silicon. Of course, stored-program computers (as the name suggests) require software in order to command and coordinate complex computations. I was fortunate to receive programming instruction from Gihan Wickramanayake (University of Colombo) and Albert Smith (Queen’s University Belfast) and their colleagues, which gave me an appreciation of how different logic gate arrays inside a computer are called into service at different stages of a run.

It took time for circumstances to change sufficiently to allow molecules to be considered as possible information processors [56]. Then it dawned on me that the 'lumophore–spacer–receptor’ system could be elaborated into Boolean logic devices with chemical inputs and luminescence output. The first device, which initiated molecular logic-based computation as an experimental field, arose in the form of a 'lumophore-spacer_1_–receptor_1_–spacer_2_–receptor_2_’ system, **3** [57], which behaved as an AND logic gate driven by Na^+^ and H^+^ inputs. In other words, luminescence emission was strong only when both Na^+^ and H^+^ were present at high levels. Each input, when supplied to the device at sufficiently high concentration, knocks out a PET pathway from its corresponding receptor to the lumophore. Even a single PET pathway disables emission. Selective receptors were the key.

**Figure 4:**
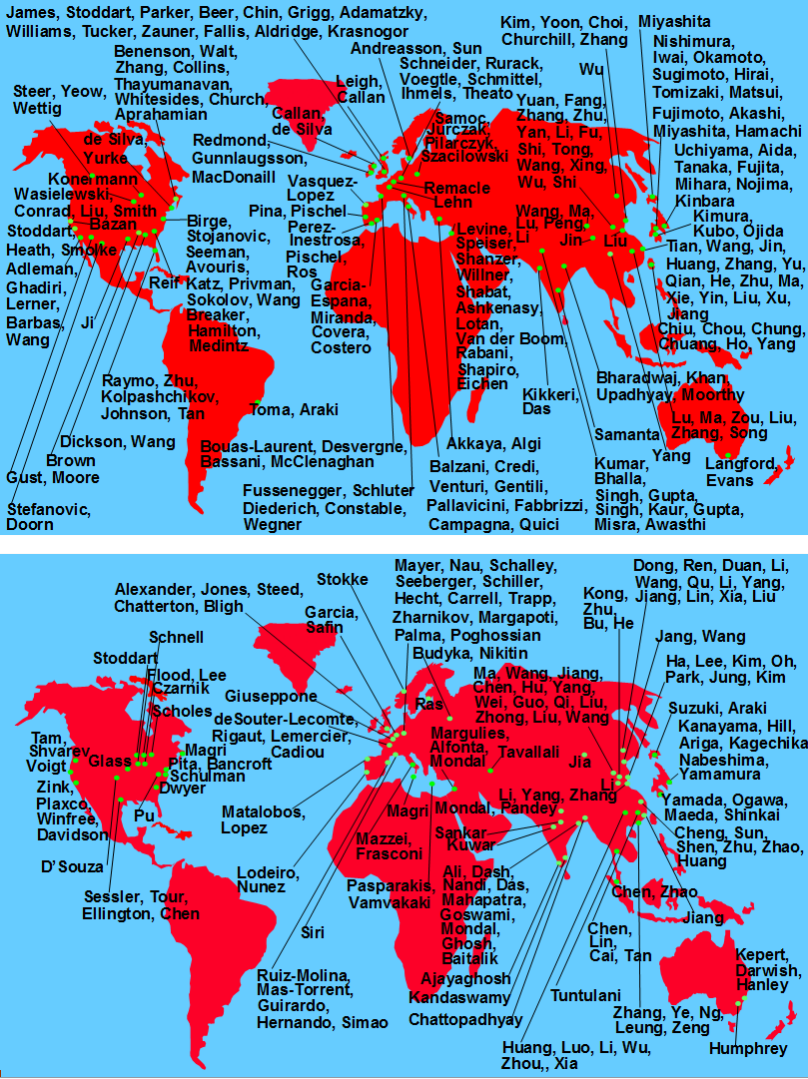
Approximate world maps of the sources of molecular logic devices. Only the names of corresponding authors from the literature are given. The simplest cases of single-input, single-output binary logic devices are not included. Adapted from [65], Copyright (2015) The Royal Society of Chemistry.

It is perhaps worth noting that a simple expansion of the 'lumophore–spacer–receptor’ system, with the attachment of an additional spacer and receptor, allowed chemistry to crossover into computer science at least in conceptual terms [16,58–59]. More generally, there was recognition that a chemical reaction (by its very name) involves a humanly noticeable response of the molecular device to some inputs such as reagents and reaction conditions. Or, in other words, chemistry is full of input–output devices based on molecules and materials. This diversity has continued to attract nearly 400 laboratories from various backgrounds and disciplines into the molecular logic field up to now (Figure 4) [16,21,60–64].

**Figure 5:**
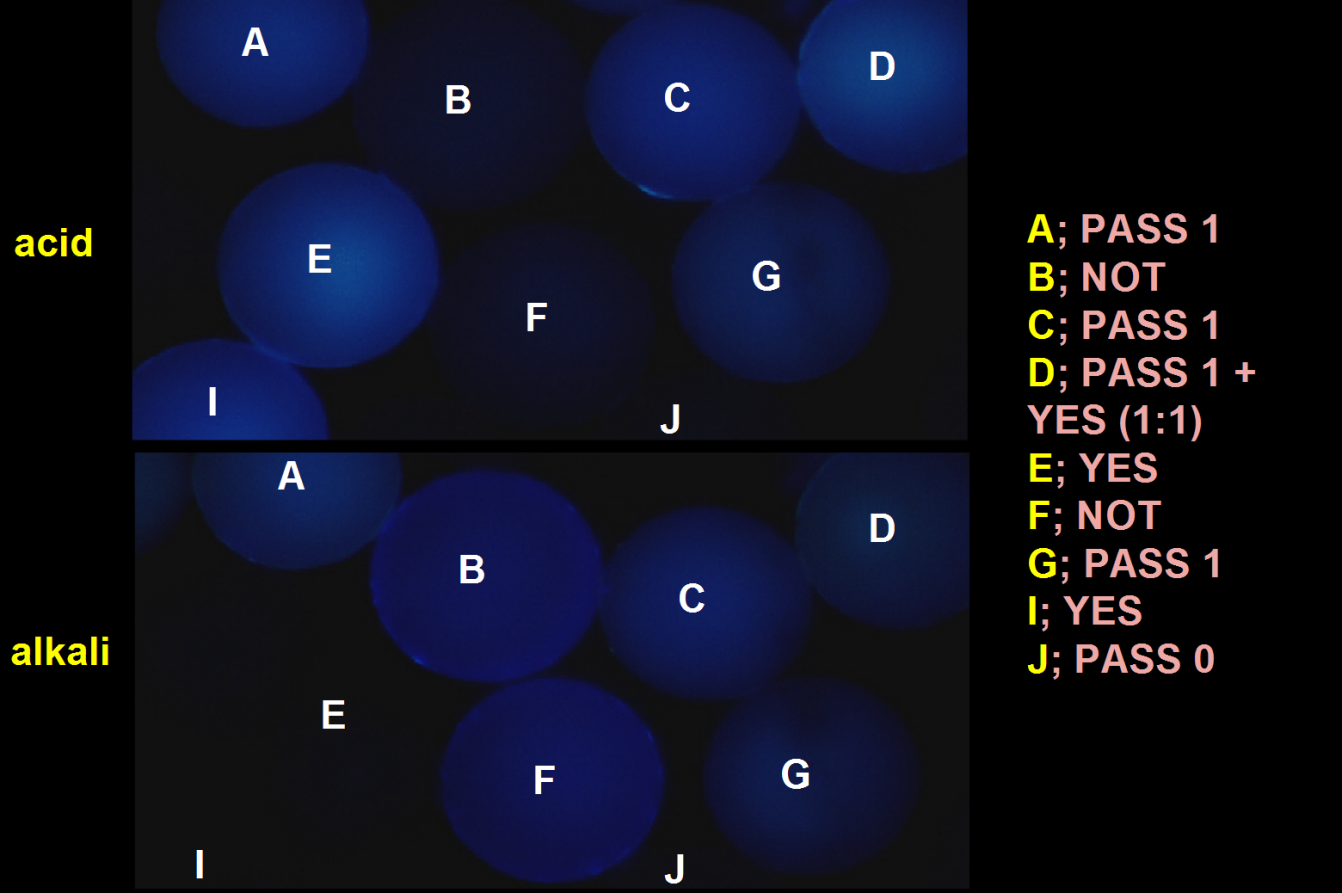
Fluorescence micrographs (excited at 366 nm) of 0.1 mm polymer beads carrying MCID tags. The beads are treated with (a) acid and (b) alkali in aqueous methanol (1:1, v/v). The logic gate type of each bead is given in the legend. Photograph adapted from [67], Copyright (2006) Macmillan Publishers Limited.

Molecular logic-based computation can be put to use in situations which benefit from the small size of fluorescent molecules. For instance, it can be useful in drug discovery. Many of these programs employ sub-millimetric polymer beads as carriers of drug candidates. These beads have to be tagged with some identification so that they can be tracked as they go through the processes of discovery and evaluation. However these beads are too small to be tagged by semiconductor-based radiofrequency identification (RFID) chips [66], which would otherwise have been the obvious choice. Molecular computational identification (MCID) tags come to the rescue [67–68]. Fluorescence colour is a useful identifier [69] but sufficient diversity is not generated in this way. Substantial diversities are created when the emission of the fluorescence colour is made conditional upon various controllable input parameters, such as the chemical nature of the input, the logic type of the response pattern and the threshold of the input concentration which triggers the response. Double tagging leads to large diversities and also gives access to multi-valued logic. The latter has a higher information content than binary versions [16,70–75], and is not error-prone under our experimental conditions of microscopic examination after washing. In contrast, multi-valued logic is very error-prone under normal conditions of computation with stored programs due to error accumulation over many computational steps. Even cases as simple as H^+^-driven YES and PASS 1 logic gates (**4** and **5** respectively) are useful as MCID tags either individually or in combination, via the acid–base control of the blue fluorescence. A YES gate produces a strong light output only when the input is present at a high level. On the other hand, a PASS 1 gate produces a strong light output whether the input is present or not. Figure 5 shows the blue fluorescence output of these and other gates on polymer beads in acid and alkaline conditions. These examples and the blood analyzer described previously (which is a Na^+^-driven YES gate with green fluorescence output) are proof that even the simplest molecular logic gates have worthwhile uses [16].

### Emulating a bit of psychology

The process of visual perception or attention protects us all everyday, by evaluating every approaching object for its threat potential [76–77]. Psychologists have found this is achieved by the retina detecting the edges of the object [78]. The physiological basis is that, within milliseconds, the image received by the rod and cone cells is passed up to the ganglion cells which can use a wiring scheme by which a group of rod and cone cells pass their responses to a single ganglion cell so that an output is eventually fired into the optic nerve only if the central rod/cone cell is illuminated while the surrounding cells are in the dark (or vice versa). The edges so extracted contain far less information than the original image so that it can be sent to the brain for quick comparison with other edges held in easily accessible memory. Once the object is identified through its edges, appropriate activation of leg muscles will enable the person to flee the scene if necessary.

Edge detection can also be achieved in a semiconductor computing context, but not with a logic gate array alone. A full stored-program computer [19–20] running edge-detection software such as the Canny algorithm [79] is required. This allows different logic arrays to be brought into action as each line in the algorithm is called out, for example. Then, light intensity gradients (or second derivatives) can be detected. Such programs are even available on mobile telephones nowadays. Such a fundamental aspect of information processing in the animal and technology worlds has also been emulated by films of bacteria, after suitable genetic modification [80], and also by reactive networks of rather high molecular-mass oligonucleotides [81], both of which involve high levels of organization in space-time.

The challenge we faced was to emulate this deep-seated animal behaviour with small molecules with no organization other than to spread them out on paper [82]. Such spreading would create a graphical user interface somewhat akin to those found in a touch-screen of a mobile telephone or in a mouse-driven screen of a stored-program computer. The treated paper could build an image after receiving a projection of the object. We do this by using a photoacid generator **6** (used to sculpt features in silicon chips) [83] in combination with a H^+^-driven ‘off-on’ fluorescent sensor **7** [84–85], which is also a H^+^-driven YES logic gate with fluorescence output. A weak pH buffer (Na_2_CO_3_) is used to poise the system in an ‘off’ fluorescent state at the beginning. Costs are kept low by employing a common two-colour ultraviolet lamp for writing (254 nm) and reading (366 nm) the information.

**Figure 6:**
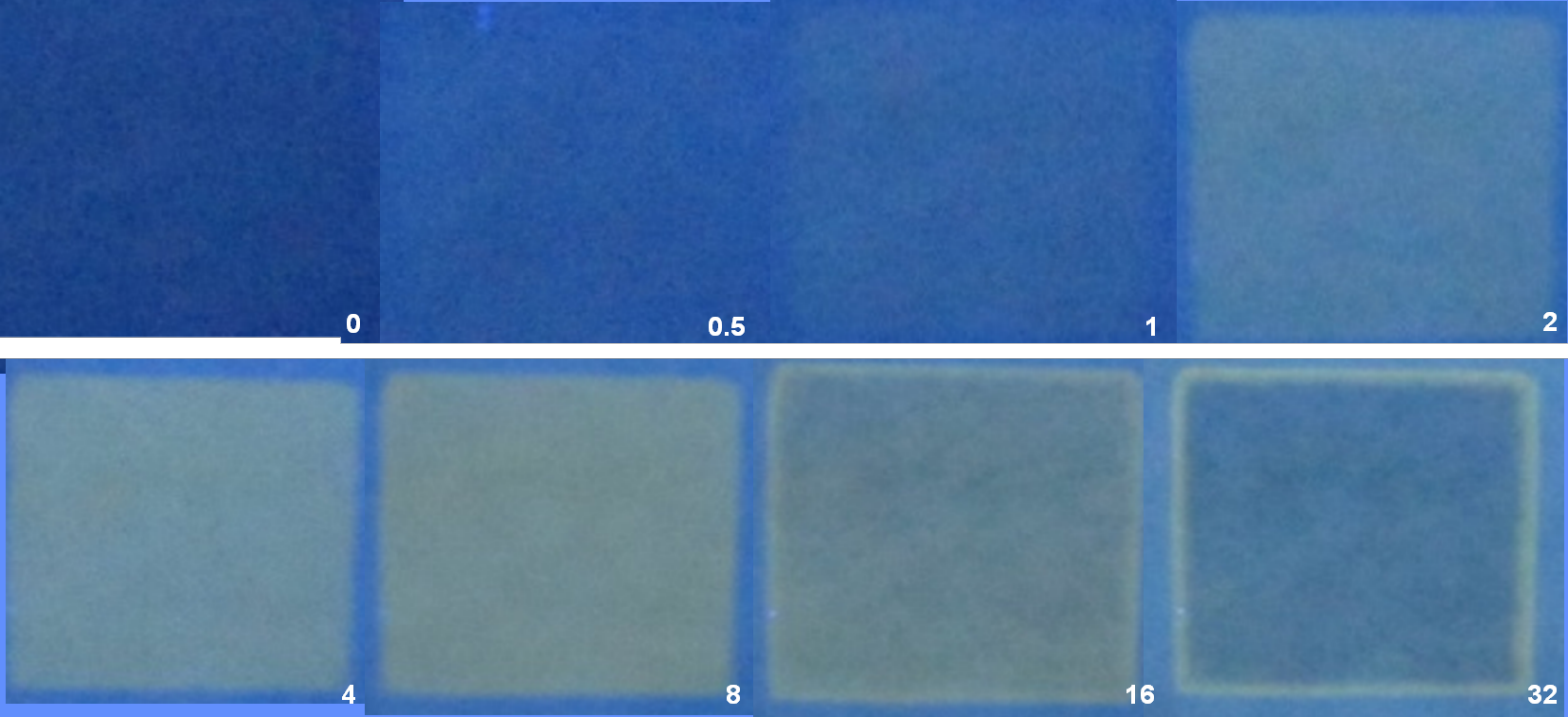
Photographs of fluorescent images (excited at 366 nm) after writing with 254 nm light through a ‘square’ mask onto the substrate, containing **6**, **7** and Na_2_CO_3_, for varying cumulative times in minutes as noted in each photograph. Scale bar = 4.0 cm. Photograph reprinted from [82], Copyright (2015) American Chemical Society.

Writing with 254 nm produces protons in the irradiated regions, which quickly overcome the weak buffer so that the fluorescence of **7** is switched ‘on’. This occurs by removal of the PET process occurring from the amine side groups to the perylenetetracarboxydiimide lumophore [84–85]. So, a positive photograph is produced at short irradiation times (Figure 6). However continued irradiation builds up the concentration of the photoproduct **8**, whose electron richness allows it to engage in a PET-based bimolecular quenching process, so that the freshly-created fluorescence is killed off again (Figure 6). This is a fluorescence ‘off–on–off’ process driven by writing light dose. ‘Off–on–off’ processes are common [86–94], with enzyme activity as a function of pH and tunnel diode current as a function of voltage being just two disparate examples [90]. ‘Off–on–off’ processes can also be understood as XOR logic behaviour [16,21] or more generally as a ternary logic function [16].

While the ‘off–on–off’ fluorescence function eventually returns most of the image to a dark state, the edges of the image remain brightly fluorescent. Diffusion of H^+^ down the gradient at the edges allows protons to outrun the lumbering **8** and create a thin region of protonated **7** which escapes the quenching. The thinness of this bright region is determined by the diffusion coefficient of H^+^ in the matrix. We dry the paper carefully so that the diffusion coefficient of H^+^ is about an order of magnitude lower than that found in bulk water [85]. This procedure results in edges of 1–2 mm thickness during an experiment runtime of about 30 minutes (Figure 6). Too much or too little drying destroys the edge detection capability quite sharply [82]. It is important to note that the edge regions are governed by much more than light dose-driven XOR logic. Indeed, the construction of serially integrated molecular logic gate arrays has required innovative schemes in the hands of several laboratories [26,95–105]. It is also sobering to realize that the parallel processing seen in the current example involves around a quadrillion molecules of the sensors to create an edge of an object about 4 cm square. What’s a quadrillion? One way to imagine this is with a bit of economics. A quadrillion dollars is around the total debt plus derivatives traded in the world (2013 figures). In contrast, the total world gross domestic product is only 70 trillion dollars.

### Emulating a bit of arts

**Figure 7:**
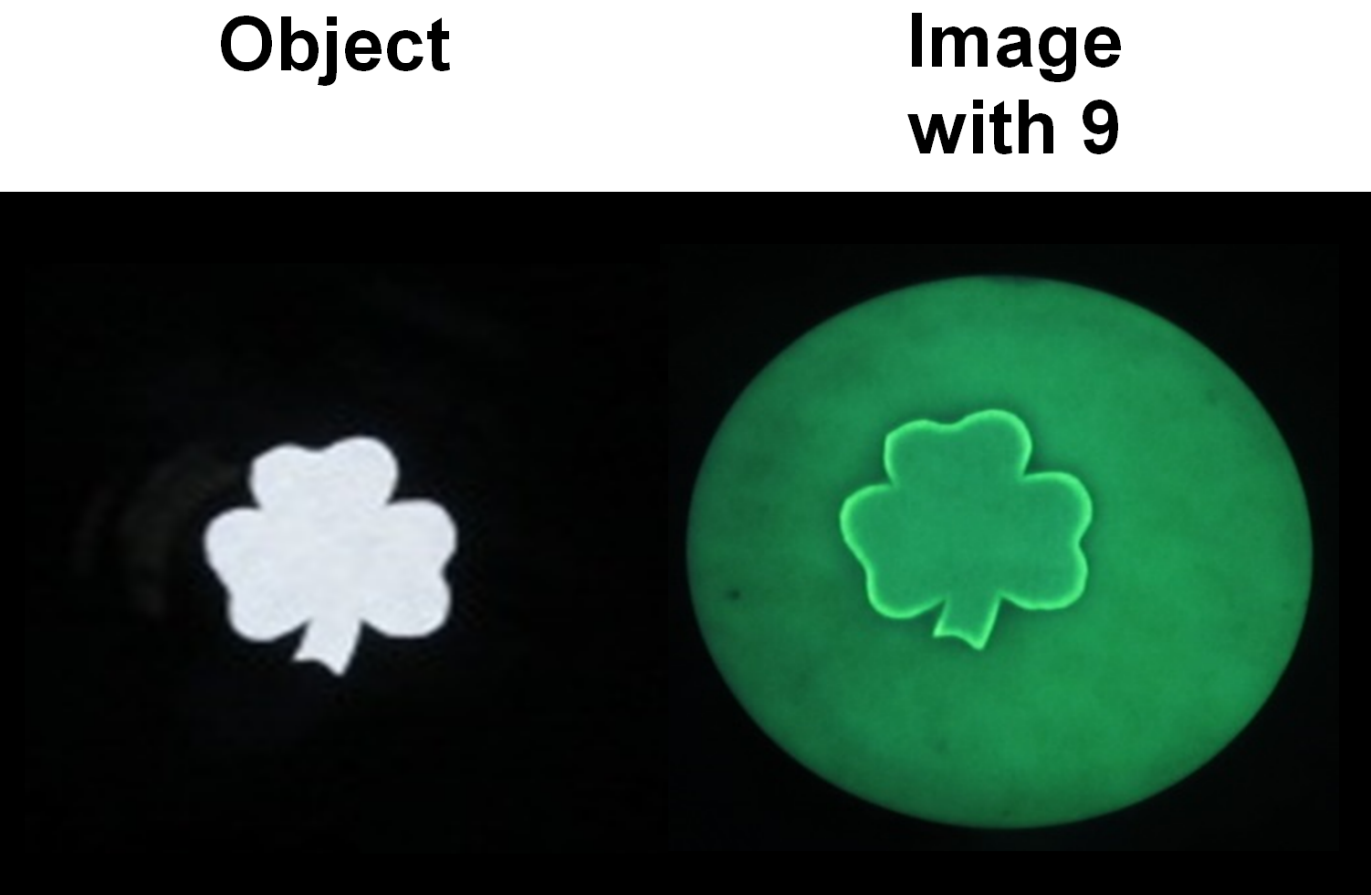
Backlit shamrock object and fluorescence image (excited at 366 nm) on paper containing **6**, **9** and Na_2_CO_3_, following writing with 254 nm light for 16 min. The filter paper diameter is 11.0 cm. Photograph reprinted from [85], Copyright (2015) The Royal Society of Chemistry.

The achievement of edge detection by small molecules allowed us to apply this idea to visual arts at a rudimentary level [85]. Outline drawing is perhaps the simplest level of sketching. Many paintings begin life as an outline drawing so that we are connecting here with a celebrated part of human culture. It seems that an outline drawing is the accurate representation of the edges detected by the artist while observing the object. Our edge-detecting composition of **9** [106], **6** and pH buffer transforms the shamrock object into a green outline suitable for a St. Patrick’s Day celebration (Figure 7). The main difference between the circumstances of Figure 6 and Figure 7 is that now the object includes arbitrarily complex curves and acute angles, as an artist would.

**Figure 8:**
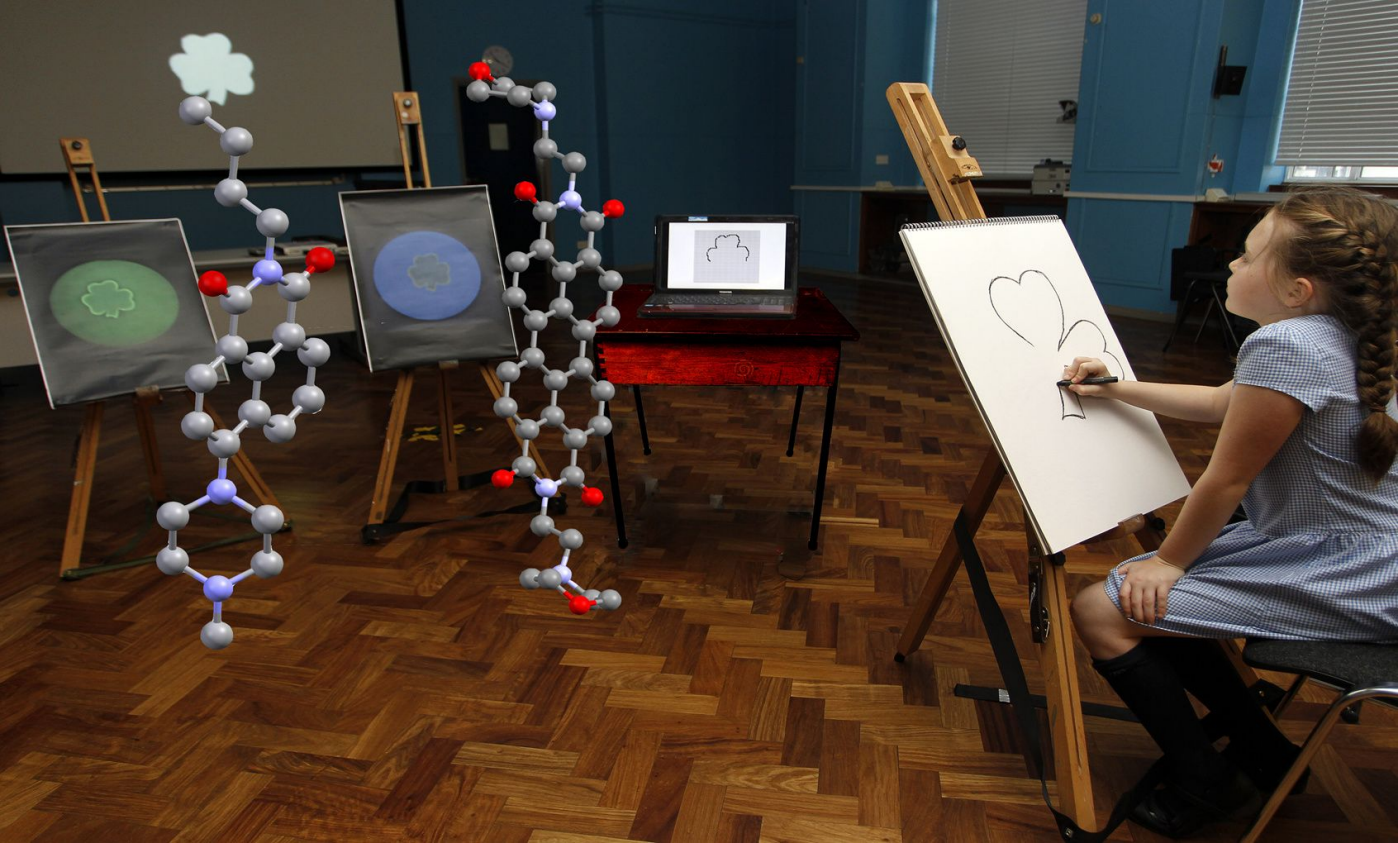
Comparison of how a child, a computer and the molecules **7** and **9** draw the outline of a shamrock object on the wall screen. Photograph used by permission of Karen-Louise Daly, Brian Daly and Aodhan O’Raghallaigh.

Figure 8 illustrates the different ways in which children, computers and molecules achieve outline drawing. Children follow the outline directly, probably by employing their edge detection ability, from an arbitrary point on the outline until the circuit is completed. Computers, running a version of the Canny algorithm [79], take an image and raster scan it so that the edge pixels emerge horizontal line by horizontal line. Thus the outline arises from a vertical stack of edge pixels. Logical molecules take an initial photographic image, expand it slightly and then erase the original photograph to leave behind the thin expansion region as the outline. This behaviour can be modelled semi-quantitatively using well-known equations of acid-base equilibria and diffusion [85].

### A bit of arts

The arts offer an excellent balancing influence for scientists. For me, one non-scientific aspect of life needs to be mentioned. Sri Lanka is a drumming nation and Northern Ireland has a very rich music tradition. I have been immensely fortunate to absorb some part of these traditions. Percussion and drumming have been an essential part of me as long as I can remember. I was fortunate to be introduced to an Irish traditional band 17 years ago and we have kept on playing since then.

### Epilogue

Since supramolecular chemistry concerns molecule–molecule interactions, its principles can be adapted to miniaturize people–people interactions or people–object interactions to the molecular level in favourable instances [107]. It is notable that such interactions may involve information transfer of some kind. Enabling molecules with the ability to gather, store, process and transmit information is a very worthwhile enterprise, especially because molecules can access important spaces where no devices but molecules may enter. Additionally, the smallness of molecules allows the mobilization of huge numbers of them in parallel so that large-area problems can be solved. The above sections have provided some examples where bright (super)molecules exploit these capabilities from a variety of contexts. I hope that younger and brighter minds will expand this variety much more. After all, there is a lot of human experience waiting to be miniaturized for useful purposes.
